# Exploring the Restoration of Brain Connectivity during Weight Normalization in Severe Anorexia Nervosa

**DOI:** 10.1192/j.eurpsy.2023.49

**Published:** 2023-07-19

**Authors:** L.-K. Kaufmann

**Affiliations:** Donders Institute for Brain Cognition and Behaviour, Centre for Cognitive Neuroimaging, Radboud University, Nijmegen, Netherlands

## Abstract

**Abstract:**

Anorexia nervosa is a persistent and often difficult to treat eating disorder with significant physical and mental health consequences. While it is known that the disorder is associated with alterations in brain functional connectivity during the phase of acute underweight, the effect of weight normalization on brain connectivity remains unclear.

This talk focuses on the recovery of intrinsic brain connectivity during weight normalization in severe anorexia nervosa, presenting data from a longitudinal study. Using resting-state functional magnetic resonance imaging, we assessed brain connectivity at three different stages of inpatient treatment. Our findings indicate that patients with severe anorexia nervosa have weaker intrinsic connectivity and altered network topology, which do not improve during treatment. These persistent disruptions in brain networks suggest that severe anorexia nervosa may have long-term effects on the way the brain processes information, even after weight is restored.

**Disclosure of Interest:**

None Declared

